# Is choroid plexus growth altered in isolated ventriculomegaly on fetal neuro-ultrasound?

**DOI:** 10.1007/s00330-024-10966-3

**Published:** 2024-07-17

**Authors:** Wei-Xi Hu, Xin Zhan, Dan Lu, Zhi-Qiang Li

**Affiliations:** 1 Department of Ultrasound in Obstetrics and Gynecology, Zhongnan Hospital of Wuhan University, Wuhan, Hubei China; 2 Department of Neurosurgery, Zhongnan Hospital of Wuhan University, Wuhan, Hubei China

**Keywords:** Prenatal choroid plexus volume altered, Isolated ventriculomegaly, Ultrasound, Prenatal diagnosis

## Abstract

**Objectives:**

Reveal developmental alterations in choroid plexus volume (CPV) among fetuses with isolated ventriculomegaly (VM) through neuro-ultrasound.

**Methods:**

This prospective study aimed to assess the development of fetal CPV in normal fetuses and those with isolated VM through neuro-ultrasound. The fetuses of isolated VM were categorized into mild, moderate, and severe groups, and subsequently, the lateral ventricle evolution was monitored. The developmental alterations in CPV among fetuses with isolated VM were determined by comparing the CPV *z*-scores with those of normal fetuses. Receiver operating characteristics curve analysis was used to assess the predictive value of altered CPV in lateral ventricle evolution.

**Results:**

A total of 218 normal fetuses and 114 isolated VM fetuses from 22 weeks to 35 weeks of gestation were included. The CPV decreased as the isolated VM was getting worse. Both fetuses with isolated moderate ventriculomegaly and those with isolated severe ventriculomegaly exhibited reduced CPV compared to normal fetuses. The CPV in fetuses with isolated mild ventriculomegaly (IMVM) varied, with some showing a larger CPV compared to normal fetuses, while others exhibited a smaller CPV. The larger CPV in cases of IMVM may serve as a predictive factor for either regression or stability of the lateral ventricle, while reduced CPV in cases of isolated VM may indicate worsening of the lateral ventricle.

**Conclusion:**

The growth volume of fetal CP exhibited alterations in fetuses with isolated VM, and these changes were found to be correlated with the evolution of the lateral ventricle.

**Clinical relevance statement:**

Neuro-ultrasound revealed varying degrees of alterations in the volume development of the choroid plexus within the fetus with isolated VM. The findings can help predict lateral ventricle prognosis, greatly contributing to prenatal diagnosis strategies for fetuses with isolated VM.

**Key Points:**

- *The volume of choroid plexus growth is altered in fetuses with isolated VM*.
- *The altered CPV in isolated VM was associated with lateral ventricle evolution*.
- *The findings are useful for prenatal counseling and managing fetuses with isolated VM*.

## Introduction

The choroid plexus (CP) is a network of blood vessels, connective tissue, and cells in the cerebral ventricular system. The CP generates cerebrospinal fluid (CSF) and regulates brain homeostasis [1]. It also participates in neuronal growth [2], day–night rhythm modulation [1], neuroimmunomodulation [3], and neurocognition [4]. CP abnormalities may affect brain homeostasis, and the impact of CP on central nervous system disorders in adults and children has been studied recently [5–8]. An increased CP volume on MRI in adults and children may be associated with psychosis spectrum disorder phenomenology and may be a specific contributor to these psychiatric conditions [7, 9].

Ventriculomegaly (VM) occurs in fetuses when the atrial width is equal to or greater than 10 mm [10] on the trans-ventricular plane of the fetal head measured using two-dimensional (2D) ultrasound [11]. It is the most common disease of the fetal central nervous system. Isolated VM is defined as VM that is not complicated by other structural abnormalities or associated with markers of aneuploidy [12]. The diameter, length, area [13], and circumference [14] of the fetal CP were measured to evaluate the separation between the CP and medial wall of the lateral ventricle [15, 16] in fetuses with isolated VM, and the “CP separating” more than 3 mm [16] may predict a poor prognosis. However, the relationship between the CP itself and fetuses with isolated VM remains unknown.

A study on 330 fetuses with isolated mild ventriculomegaly (IMVM) demonstrated that 7.9% of the cases had a neurodevelopmental delay after birth [17]. The incidence of neurodevelopmental delay in moderate unilateral or bilateral VM varies between 7% [18] and 12% [19]. Carta et al [20] reported that approximately 60% of fetuses with isolated severe bilateral ventriculomegaly presented abnormal neurodevelopment after birth. Further investigation is needed to determine if the CP can be used as an additional marker for distinguishing cases of fetuses with isolated VM who later develop neurodevelopmental disorders. Firstly, we will examine any developmental changes in the CP itself in fetuses with isolated VM, aiming to provide valuable insights as a potential marker for future research on neurodevelopmental disorders.

The *z*-score model [21], introduced in prenatal diagnosis for ultrasound, has demonstrated its validity as an accurate assessment method for evaluating the developmental changes of fetal organs. The objective of this investigation was to examine the developmental alterations in CP volume (CPV) among fetuses with isolated VM using neuro-ultrasound.

## Material and methods

### Study population

The study was performed between June 2022 and June 2023 in the fetal medicine unit of Wuhan University Zhongnan Hospital. This observational study was carried out following the “Strengthening the Reporting of Observational Studies in Epidemiology (STROBE)” standards (Supplementary Material, Table 1).

Normal fetuses and fetuses diagnosed with isolated VM, between 22 weeks and 35 weeks of gestation, underwent neuro-ultrasound examination and were randomly enrolled in this study. The following data was gathered during the neuro-sonography assessment: fetal biparietal diameter (BPD), gestational week (GA), and sides of CP (left or right). GA was assessed by reviewing the fetal crown–rump length during an early pregnancy ultrasound (between 11 weeks and 14 weeks). Following neuro-sonography, the lateral ventricular width of each fetus with isolated VM was monitored every 2 weeks until delivery. The pregnancy outcomes of fetuses, including birth weight (BW), week of birth, newborn gender, and Apgar Score, were meticulously documented. The clinical information on pregnant mothers and their fetuses was obtained through telephone interviews and the computerized patient records of Zhongnan Hospital, Wuhan University. The local institutional review committee approved this study (Research Ethics Committee reference number: 2022123 K), and all participants provided informed consent.

### Subjects and subgroup definitions

The control group included singleton pregnancies with normal fetuses, each having two atrial width estimates of less than 10 mm. The study group inclusion criteria were: (1) singleton pregnancies with isolated VM fetuses; (2) fetuses with at least one or two atrial width measurements of 10 mm or more [10]; (3) absence of other structural anomalies or syndromes (e.g., intracranial hemorrhage); and (4) confirmation of no intracranial structural abnormalities through postnatal MRI. The exclusion criteria for all groups included: (1) mothers who smoked or drank alcohol; (2) mothers or fetuses with medical conditions that may have an impact on fetal development or growth (e.g., diabetes, hypertension, small for gestational age (GA) fetus, intrauterine growth restriction); (3) mothers with medical conditions requiring treatment or medication during pregnancy (e.g., coronavirus disease 2019 or Influenza virus infection); (4) fetal chromosomal abnormalities or fetal intrauterine infection; (5) abortion or termination of pregnancy; and (6) low-quality 3D images of fetal CP.

According to the inclusion and exclusion criteria, a total of 114 fetuses diagnosed with isolated VM and 218 normal fetuses, were ultimately included in this study. The study group was divided into three categories based on the atrial width of fetuses [10]: IMVM (10–12 mm), isolated moderate ventriculomegaly (IMODVM) (13–15 mm), and isolated severe ventriculomegaly (ISVM) (> 15 mm). Based on the prognosis of fetal atrial width during pregnancy, the study cohort was further stratified into three subgroups within each category [22]: regression (atrial width decrease exceeding 3 mm [22]), stable (no significant change in atrial width beyond 3 mm [22]), and progression (atrial width increase of more than 3 mm [22]).

### Fetal neuro-ultrasound examinations

**Fig. 1:**
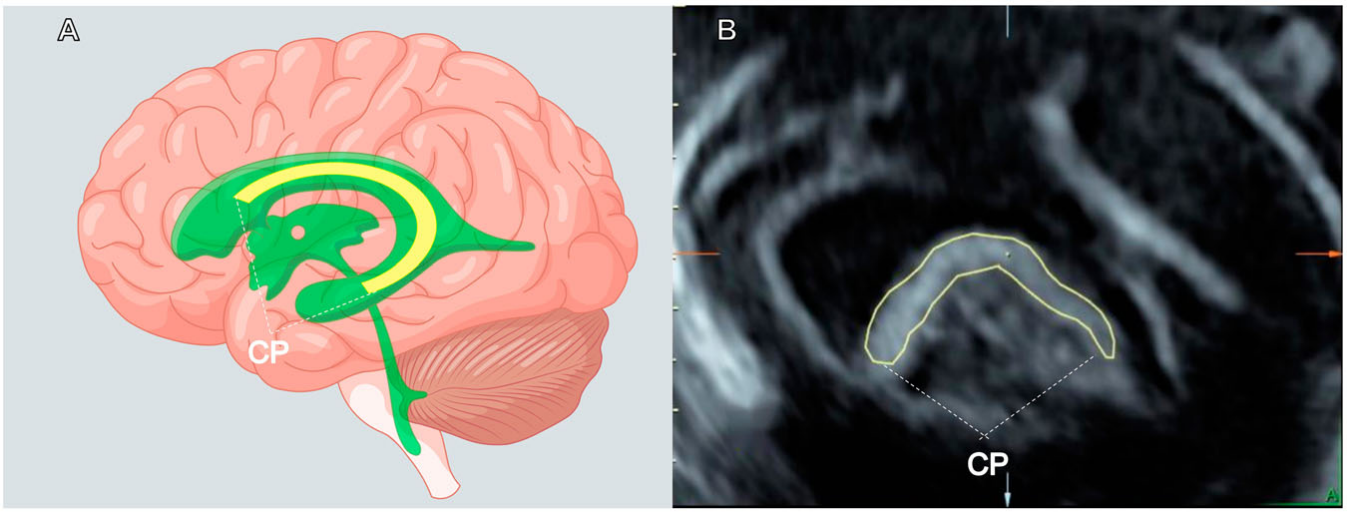
Determination of section for CP assessment (**A** By Figdraw; **B** By ultrasound), the three-dimensional images of fetal CP were acquired from the parasagittal plane of the fetal head

Neuro-ultrasound scanners employing the Voluson E8 or E10 ultrasound system (General Electric Healthcare Ultrasound, Zipf, Austria) equipped with a 2–6 MHz RM6C transducer were utilized for fetal brain image acquisition. Participating mothers underwent a transabdominal ultrasound examination, and the transducer was positioned in the fetal brain. The steps for obtaining ultrasonic images are as follows:

1. Trans-ventricular plane acquisition [11]: the probe was positioned on the brain to horizontally expose and align its midline, ensuring a clear cross-sectional view. Subtle adjustments were made to enhance the visualization of both the septum pellucidum and posterior horn of the lateral ventricle.
2. 3D images of CP acquisition: the probe was rotated 90° to capture the median sagittal section of the fetal brain using the trans-ventricular plane. By tilting the probe slightly on either side of the head, we could clearly visualize CP in the lateral ventricle and obtain a parasagittal plane [23] of the fetal brain (Fig. 1). We activated 3D ultrasound mode and obtained 3D images of CP.

**Fig. 2:**
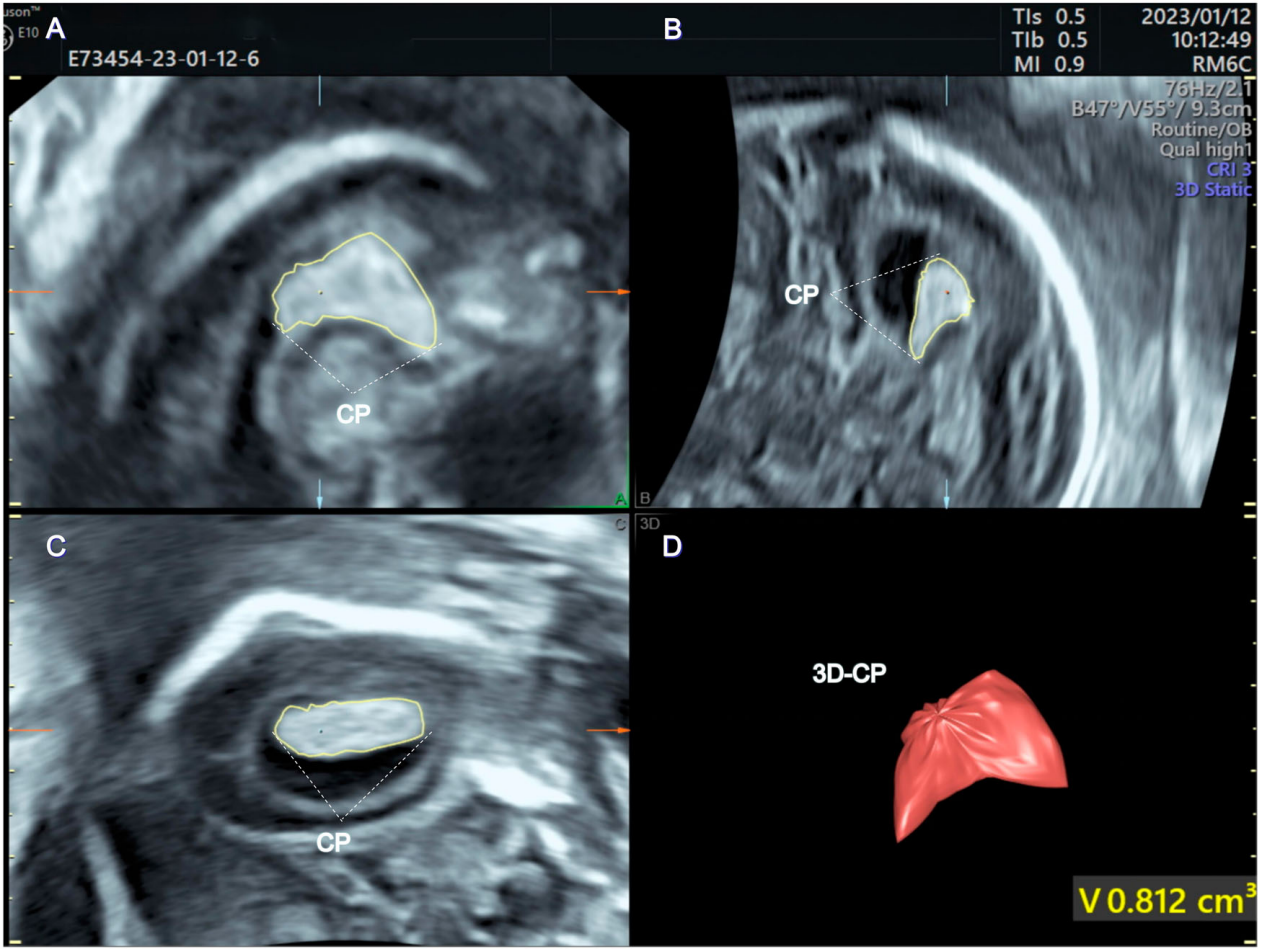
Methods for calculating the volume. Tracing the CP area in the lateral ventricle with rotational steps of 30° (**A**, **B**, **C**), the CP volume is automatically measured by software (**D**)

The images were stored in ultrasonic instruments and workstations for subsequent measurement and post-processing. The measurement steps were as follows:

1. Lateral ventricle width [11]: on the trans-ventricular plane, calipers were positioned within the echoes generated by the lateral ventricle, specifically at the level of the parietooccipital sulcus and perpendicular to the ventricular cavity [11].
2. CP volume [24]: using [24, 25] virtual organ computer-aided analysis (VOCAL) software (General Electric Healthcare, Zipf, Austria), tracing the CP area in the lateral ventricle with rotational steps of 30° as Ritsuko et al [24] previously described, and then CP volume was automatically measured (Fig. 2).

The acquired images were independently analyzed and measured offline by two experienced sonographers, who were blinded to the clinical information of the fetuses. The average of the two measurements was considered as the final value for each case and subsequently reviewed by senior specialists.

### Statistical analysis

The quantitative data were summarized as mean (standard deviation, SD) or median (interquartile range, IQR). The categorical variables were represented as percentages (%). The inter-observer agreement between sonographers’ measurements was assessed using intra-class correlation coefficients (ICC). Before statistical analysis, the best-fitted *z*-score models [21] for CPV in controls. The correlation between fetal parameters (GA, BPD) and CPV was examined, and the most suitable equations were selected to establish the *z*-score model as DeVore et al [21] described. CPV *z*-scores in controls were calculated and the Shapiro–Wilk test was used to verify data normality. ALL CPV measurements were transformed into *z*-scores using the formula: *z*-scores = (observed measurement − predicted value)/[1.25 × (observed measurement − predicted value)].

*t*-Tests or Mann–Whitney *U*-tests were employed to analyze the independent variables influencing CPV or CPV *z*-scores for comparing two groups, while Kruskal–Wallis tests or analysis of variance (*F*-test) were utilized for comparisons involving more than two groups. The Spearman correlation analysis and linear regression were used to value the relationship between independent variables (lateral ventricular width and birth weight) and dependent variables (CPV, CPV *z*-scores) in both the control group and study group. In the study group, receiver operating characteristic (ROC) curves were employed to investigate the predictive value of CPV *z*-scores in relation to lateral ventricular evolution. IBM SPSS statistics software (version 26.0.0.0; SPSS Inc.) was used to analyze the data. Statistical significance was set at *p* < 0.05.

## Results

The study included 224 normal fetuses, with an equal number of left and right CP cases (224 each). However, 58 CP cases were excluded due to poor image quality. Finally, the control group consisted of 390 CP cases (199 left CP and 191 right CP) from 218 normal fetuses. The study group consisted of 114 fetuses with isolated VM, all exhibiting high image quality. This included 89 fetuses with unilateral isolated VM (58 left CP, 31 right CP) and 25 fetuses with bilateral isolated VM (25 left CP, 25 right CP). Ultimately, the study group consisted of a total of 139CP. The CPV and atrial width measurements showed excellent agreement between the two sonographers. The ICC for CPV measurements were 0.84 (*p* < 0.01) and 0.79 (*p* < 0.01) for the study group and control group, respectively. Additionally, the atrial width measurements exhibited values of 0.93 (*p* < 0.01) and 0.85 (*p* < 0.01) for the study group and control group. Table 1 presents the demographic and clinical characteristics as well as pregnancy outcomes of the participating fetuses. The mean GA and BPD did not differ significantly among the control, IMVM, and IMODVM groups (all *p* > 0.05). The fetuses were all delivered without any complications, and their Apgar scores fell within the normal range.

**Table 1:** Maternal and fetal characteristics in 139 CP from the study group and 390 CP from the control group

	Controls, (*n* = 390)	Study group, (*n* = 139)		
		IMVM, (*n* = 102)	IMODVM, (*n* = 29)	ISVM, (*n* = 8)
Maternal characteristic				
Age (years)	33.54 ± 3.61**	32.06 ± 5.11	32.17 ± 5.61	35.00 ± 6.21**
GA at assessment (weeks)	28.47 ± 4.13**	28.11 ± 4.03	28.10 ± 2.27	32.50 ± 1.58**
Fetal characteristics				
BPD at assessment (cm)	7.11 ± 1.09**	7.06 ± 1.11	7.54 ± 0.63	9.05 ± 0.23**
Lateral ventricle width (mm)				
Left	7.5 ± 1.50**	10.53 ± 0.62**	13.27 ± 0.46**	15.50 ± 0.71**
Right	7.2 ± 1.30**	10.71 ± 0.80**	13.14 ± 0.36**	15.50 ± 0.58**
CPV (mm^3^)	813.500 (246.250)**	909.500 (511.000)**	608.621 ± 78.425**	667.500 ± 64.41**
CPV *z*-scores				
Against GA	− 0.052 ± 0.94**	2.563 (6.577)**	− 2.600 (2.11)**	− 4.46 ± 0.98**
Against BPD	0.001 ± 0.97**	2.170 (5.438)**	− 3.590 (1.81)**	− 4.86 ± 0.93**
Isolated VM, *n* (%)				
Lateral ventricle evolution				
Regression	–	35 (34.31)	0 (0)	0 (0)
Stable	–	55 (53.92)	17 (58.62)	8 (100)
Progression	–	12 (11.76)	12 (41.38)	0 (0)
Pregnancy outcome				
Apgar score	8.72 ± 1.50	8.4 ± 1.1	8.2 ± 1.6	7.9 ± 0.6
Birth weight (g)	3217 ± 244	3421 ± 436	3155.5 ± 405	3442.5 ± 376
Birth week (weeks)	37.41 ± 1.90	37.44 ± 1.82	37.11 ± 2.06	36.75 ± 1.26

Data are given as mean ± SD or median (interquartile range) or *n* (%)

*VM* ventriculomegaly, *IMVM* isolated mild ventriculomegaly, *IMODVM* isolated moderate ventriculomegaly, *ISVM* isolated severe ventriculomegaly, *CP* choroid plexus, *CPV* choroid plexus volume, *GA* gestational age, *BPD* biparietal diameter, *SD* standard deviation

**Means there was a difference between groups, and *p* < 0.01

### *z*-Score modeling in CPV in controls

**Fig. 3:**
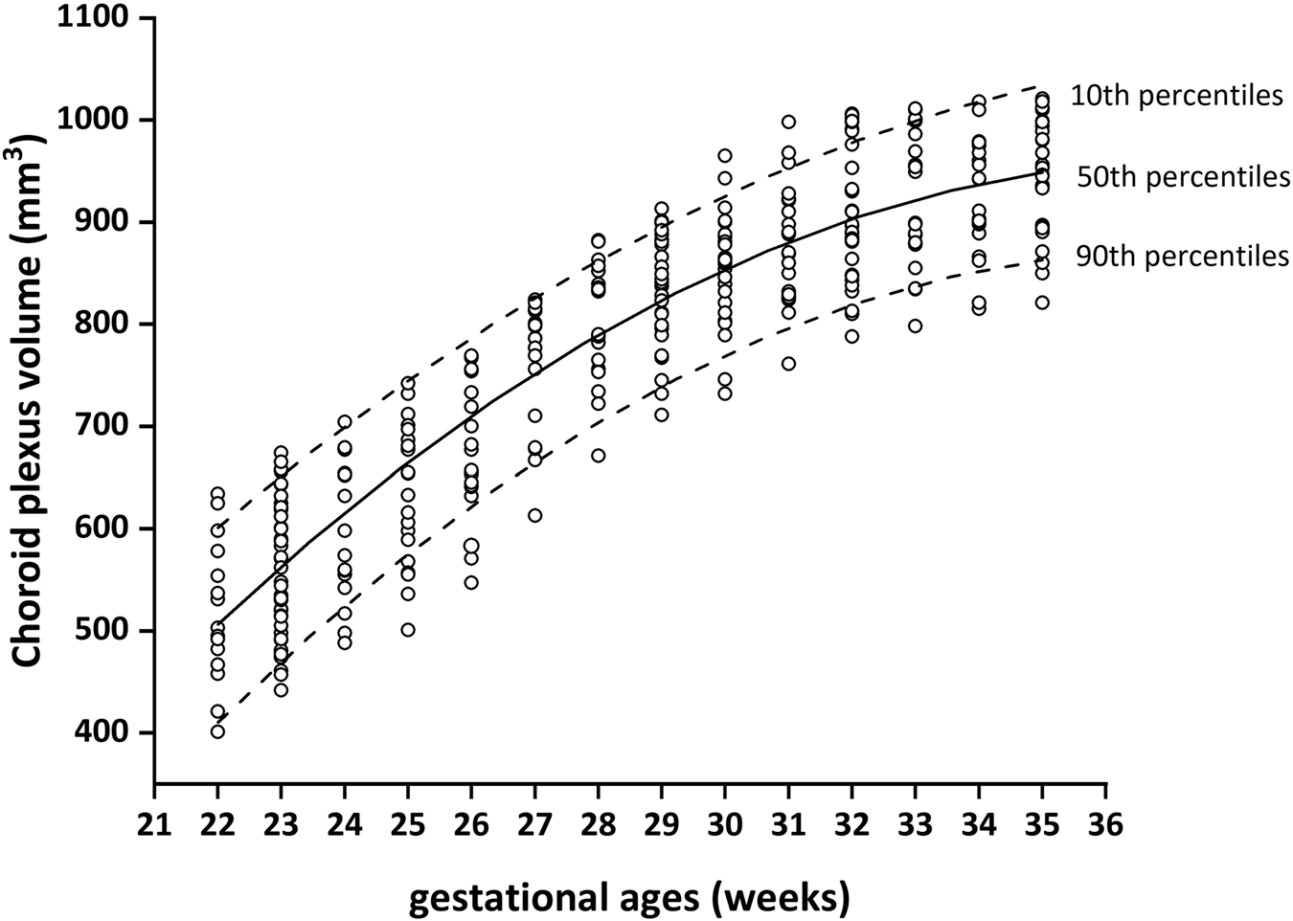
Scatter plot showing reference range for 390 CPV according to GA in 218 normal fetuses

The data presented in Fig. 3 demonstrates a rapid increase in CPV by over half of its initial volume between 22 GA and 28 GA, followed by a deceleration in growth rate from 29 GA to 35 GA. The reference values for the CPV for each GA are listed in Supplementary Material (Table 2). The CPV showed a positive correlation with GA or BPD, and quadratic curve regression analysis revealed highly significant associations between them (*p* < 0.01). The inherent collinearity between GA and BPD prompted us to develop separate *z*-score models based on GA and BPD. The equations we created were efficient and could forecast the CPV normality curve (Supplementary Material, Table 3 and Fig. 1).

### Comparison in CPV *z*-scores

In both the control and study groups, the analysis of factors influencing CPV and CPV *z*-scores revealed no significant correlations with the following variables (Table 2 and Supplementary Material, Table 4): side of CP (left or right), neonatal gender (male or female), and birth weight. The control group revealed a significant positive correlation between both CPV and CPV *z*-scores and an increase in lateral ventricle width (all *p* < 0.01). However, the study group revealed a gradual decrease in both CPV and CPV *z*-scores with increasing lateral ventricular width (all *p* < 0.01). The Spearman correlation analysis and line analysis were performed in Supplementary Material, Tables 4 and 5. The IMVM fetuses had higher *z*-scores compared to the control group, while IMODVM fetuses and ISVM fetuses showed lower *z*-scores than the controls (all *p* < 0.01, Table 1). And *z*-scores in IMODVM were smaller than IMVM but larger than ISVM (both *p* < 0.01, Table 1).

**Table 2:** The univariate analysis examined the factors that influence CPV or CPV *z*-scores separately in the control group, IMVM fetuses, and IMODVM fetuses

Parameters	Number of CP	CPV, (mm^3^)	*p* value	CPV *z*-scores against GA	*p* value	CPV *z*-scores against BPD	*p* value
Control group							
Left CP	199	821 (244.000)	0.198	− 0.05 ± 0.93	0.995	− 0.00 ± 0.94	0.952
Right CP	191	811 (263.000)		− 0.05 ± 0.97		0.00 ± 1.00	
Female newborn	186	812 (237.000)	0.291	− 0.11 ± 0.99	0.239	− 0.04 ± 1.00	0.369
Male newborn	204	822 (253.500)		0.00 ± 0.90		0.04 ± 0.93	
IMVM fetuses							
Left CP	64	840.500 (509.5)	0.082	2.936 (5.418)	0.093	2.890 (4.991)	0.124
Right CP	38	757.500 (587)		0.899 (6.861)		0.799 (6.202)	
Male newborn	56	952.000 (495.000)	0.264	2.946 (5.716)	0.095	2.890 (5.352)	0.127
Female newborn	46	836.500 (454.250)		1.414 (6.384)		1.025 (5.702)	
IMODVM fetuses							
Left CP	15	602.27 ± 61.11	0.66	− 2.91 ± 1.40	0.634	− 3.26 ± 1.58	0.473
Right CP	14	615.43 ± 95.55		− 2.71 ± 0.69		− 3.65 ± 1.29	
Male newborn	16	600.13 ± 51.95	0.556	− 3.03 ± 1.20	0.261	− 3.41 ± 1.29	0.876
Female newborn	13	619.08 ± 103.75		− 2.56 ± 0.95		− 3.50 ± 1.65	

Data are given as mean ± SD or median (interquartile range)

*VM* ventriculomegaly, *IMVM* isolated mild ventriculomegaly, *IMODVM* isolated moderate ventriculomegaly, *ISVM* isolated severe ventriculomegaly, *CP* choroid plexus, *CPV* choroid plexus volume, *GA* gestational age, *BPD* biparietal diameter, *SD* standard deviation

*p* < 0.01 Means there was a difference between groups

**Fig. 4:**
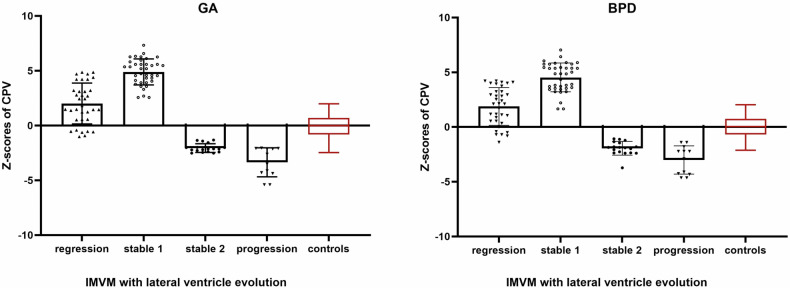
The *z*-scores against gestational age (GA) or biparietal diameter (BPD) in IMVM with lateral ventricle evolution. The evolution of the lateral ventricle was divided into regression, stable (stable1 and stable2), and progression

In IMODVM, the significantly smaller *z*-scores were found to be associated with the progression of lateral ventricles (*p* < 0.01, Table 3). In IMVM, lateral ventricle regression was observed when their *z*-scores slightly exceeded those of controls, stable lateral ventricles were noted when their *z*-scores significantly surpassed those of controls, and fetuses with progressive lateral ventricles showed significantly lower *z*-scores (all *p* < 0.01, Table 3). The scatter plot analysis (Fig. 4) revealed two distinct datasets of *z*-scores in IMVM fetuses regarding lateral ventricle stability prognosis. We further divided the stable subgroup into two subgroups: stable1 (with *z*-scores greater than zero) and stable2 (with *z*-scores less than zero). The stable1 subgroup showed significantly higher values than controls and other IMVM, while the stable2 subgroup exhibited significantly lower values than controls but higher than progressive IMVM (all *p* < 0.01, Table 3). The prognostic characteristics of the fetal lateral ventricle in the study group are shown in Table 4. The linear regression analysis (Supplementary Material, Table 5) revealed that higher CPV *z*-scores were associated with slower regression of atrium width in IMVM with regressive lateral ventricular (*p* < 0.01). The available data from other groups were insufficient to facilitate further linear regression analysis.

**Table 3:** Differences in fetal CPV *z*-scores were examined by stratifying fetuses into subgroups based on their lateral ventricular prognosis

Groups of fetuses	Number of CP	CPV *z*-scores against GA	*p* value	CPV *z*-scores against BPD	*p* value
Prognosis of lateral ventricular width in IMVM					
Regression	35	2.01 ± 1.86**	< 0.001	1.88 ± 1.72**	< 0.001
Stable	55	4.05 (7.47)**		3.54 (6.81)**	
Stable1 subgroup	37	4.89 ± 1.18**		4.53 ± 1.32**	
Stable2 subgroup	18	− 2.06 ± 0.39**		− 1.94 ± 0.63**	
Progression	12	− 3.35 ± 1.32**		− 3.01 ± 1.29**	
Prognosis of lateral ventricular width in IMODVM					
Stable	17	− 2.02 ± 0.64**	< 0.001	− 2.81 ± 1.56**	< 0.001
Progression	12	− 3.94 ± 0.40**		− 4.36 ± 0.38**	

Data are given as mean ± SD or median (interquartile range); *p* < 0.01 means there was a difference between groups

*VM* ventriculomegaly, *IMVM* isolated mild ventriculomegaly, *IMODVM* isolated moderate ventriculomegaly, *CP* choroid plexus, *CPV* choroid plexus volume, *GA* gestational age, *BPD* biparietal diameter, *SD* standard deviation

**Means was a difference between the subgroups, with *p* < 0.01

**Table 4:** The prognostic characteristics of the fetal lateral ventricle in the study group

Groups of study group	Prognostic classification for the lateral ventricle	Initial atrial width, (mm)	Final atrial width, (mm)	GA at initial ultrasound, (weeks)	GA at final change in atrial width, (weeks)	Time interval, (weeks)
IMVM	Regression	10.57 ± 0.70**	7.57 ± 0.70**	27.89 ± 3.67	31.09 ± 4.23	3.20 ± 1.23
IMVM	Stable1	10.57 ± 0.56	10.81 ± 1.79	28.95 ± 4.40	–	–
IMVM	Stable2	10.44 ± 0.78	10.44 ± 1.50	27.22 ± 3.81	–	–
IMVM	Progression	11.00 ± 0.85**	14.17 ± 0.84**	27.50 ± 4.21	31.83 ± 2.86	4.33 ± 1.67
IMODVM	Stable	13.35 ± 0.49	14.77 ± 0.83	27.12 ± 2.12	–	–
IMODVM	Progression	13.00 ± 0.000**	16.00 ± 0.00**	29.50 ± 1.73	32.50 ± 2.02	3.00 ± 1.04
ISVM	Stable	15.17 ± 0.41	15.50 ± 0.55	32.50 ± 1.58	–	–

Data are given as mean ± SD

*IMVM* isolated mild ventriculomegaly, *IMODVM* isolated moderate ventriculomegaly, *ISVM* isolated severe ventriculomegaly, *GA* gestational age, *SD* standard deviation

**Means was a difference between the groups, with *p* < 0.01

### Prediction in lateral ventricle prognosis

The CPV *z*-scores exhibited excellent accuracy in predicting the prognosis of lateral ventricles during ROC curve analysis, with their AUC ranging from 0.72 to 1 (Table 5). The *z*-scores below − 3.23 (GA as reference) or below − 3.68 (BPD as reference) in IMODVM indicated progressive lateral ventricle enlargement. In the IMVM fetuses, z-scores below − 2.56 (GA as reference) or lower than − 2.64 (BPD as reference) indicated progression of the lateral ventricle, while *z*-scores ranging from − 1.12 to 3.25 (GA as reference) or − 0.94 to 3.07 (BPD as reference) indicated regression of the lateral ventricle. The lateral ventricle would remain stable if *z*-scores were above 3.25 (with GA as a reference) or higher than 3.07 (with BPD as a reference). Similarly, *z*-scores between − 2.56 and − 1.12 (GA as reference) or between − 2.64 and − 0.94 (BPD as reference) indicate the stability of the lateral ventricle.

**Table 5:** ROC curves for CPV *z*-scores of predictive value in lateral ventricle evolution in the study group

Predicted parameter	Control parameter	AUC, (95% CI)	Youden’s index	Predicted criterion	Sensitivity, (%)	Specificity, (%)	*p* value
Prognosis of lateral ventricular width in IMVM							
CPV *z*-scores against GA							
Regression	Stable1	0.89	168.03	< 3.25	77.14	91.89	< 0.01
Regression	Stable2	1	199	> − 1.12	100	100	< 0.01
Progression	stable2	0.76	157.33	< − 2.56	58.33	100	< 0.01
CPV *z*-scores against BPD							
Regression	stable1	0.87	162.32	< 3.07	71.43	91.89	< 0.01
Regression	stable2	0.99	196.14	> − 0.94	97.14	100	< 0.01
Progression	stable2	0.72	143.44	< − 2.64	50	94.44	< 0.01
Prognosis of lateral ventricular width in IMODVM							
CPV *z*-scores against GA							
Progression	stable	1	199	< − 3.23	100	100	< 0.01
CPV *z*-scores against BPD							
Progression	stable	0.94	193.12	< − 3.68	100	94.12	< 0.01

*VM* ventriculomegaly, *IMVM* isolated mild ventriculomegaly, *IMODVM* isolated moderate ventriculomegaly, *CPV* choroid plexus volume, *GA* gestational age, *BPD* biparietal diameter, *AUC* areas under the receiver operating characteristic, *ROC* receiver operating characteristic

## Discussion

### Main findings

The study used neuro-ultrasound technology to demonstrate developmental changes in fetal CPV. Firstly, it showed an increase in CPV development from 22 to 35 GA in normal fetuses. Secondly, it found that fetuses with IMVM had higher CPV compared to normal ones, while those with IMODVM, ISVM, and some IMVM had lower CPV. Thirdly, increased CPV in IMVM may indicate regression or stability of the lateral ventricle, while decreased CPV in both IMVM and IMODVM may suggest worsening of the lateral ventricle.

### Comparison with other studies

Although fetal ultrasound typically begins at 18 GA during the second trimester, some mothers choose ultrasounds beyond this timeframe. Obtaining precise 3D images of CP becomes increasingly challenging after 35 GA. As a result, the final cohort of fetuses included in this observational study ranged from 22 to 35 GA. In controls, a gradual increase in CPV was observed as GA advanced. We first discovered that CPV increased rapidly from 22 to 28 weeks of GA in normal fetuses, and then the growth rate slowed from 29 weeks to 35 weeks of GA. To analyze the relationship between CPV values while accounting for the influence of GA and BPD, *z*-score models were established following previous methods [21]. The CPV and CPV *z*-scores all showed significant positive correlations with atrial width. Approximately 80–90% of CSF [1] is synthesized by the CP within the lateral ventricles. Pawar et al [26] and Hubert et al [27] reported that a larger CPV in adults increased CSF production. We suggested that the larger CPV in normal fetuses results in an increase in CSF production, leading to an enlargement of the lateral ventricular width.

Previous studies described the atrophic CP [28] in ISVM fetuses. However, the causal relationship [29] between the abnormality of CP and hydrocephalus remained inconclusive. We found that the study group exhibited a negative correlation between CPV and increased atrial width, indicating a gradual decrease in CPV with worsening isolated VM severity. We observed fetuses with IMODVM, ISVM, and certain IMVM exhibited smaller CPV compared to normal fetuses; a novel finding was that some fetuses with IMVM displayed larger CPV. Previous research had focused on the separation [15, 16] between CP and lateral ventricle, they [15, 16] believed that a greater CP separation was positively correlated with the probability of lateral ventricle progression. Our findings showed that there was a significant correlation between smaller CPV and lateral ventricle progression. The CP hemorrhage and epithelial cell death had been observed in mice with hydrocephalus [30]. What’s more, damage to the CP [31] may lead to increased permeability of CP epithelial cells and blood vessels, increased CSF volume, and VM aggravation. We hypothesized that the smaller size of the CPV in isolated VM suggests that the CP structure and function have already been destroyed, leading to a subsequent continuous increase in CSF production and lateral ventricular width.

The IMVM group revealed an intriguing phenomenon in which fetuses with slightly larger CPV than normal fetuses exhibited a subsequent regression in the lateral ventricle, while those with much larger CPV maintained a stable lateral ventricular. We postulated that a slight increase in CPV could potentially represent a normal variation, leading to transient lateral ventricles dilation. We further hypothesized that once equilibrium is restored in CSF absorption and release, there would be a reduction in CSF, concomitant with a decrease in lateral ventricles width. Additionally, fetuses with IMVM and an expected regression in lateral ventricle width had a longer duration of regression when their CPV was larger.

We inferred that the significantly enlarged CP we observed in IMVM with stable lateral ventricles did not conform to the standard variant, suggesting that it may have resulted in an increase in CSF and subsequently maintained the width of lateral ventricles. Forte et al [32] reported a rare case of CP hyperplasia in fetal with severe VM, characterized by significantly enlarged CP, wherein progressive enlargement of the lateral ventricle was observed. The CPV size in our study may not have been sufficiently large to significantly increase atrial width. In addition, if the CPV in the IMVM group were slightly smaller than that in the controls, the lateral ventricle width would also remain unchanged. As previously mentioned, a significantly smaller CPV in isolated VM may indicate disruption of the CP structure and function, leading to a sustained increase in CSF and progression of lateral ventricles. The slightly smaller CPV in IMVM fetuses may suggest that the impairment of CP function and structure is not significant enough to induce further CSF accumulation, thereby maintaining a stable lateral ventricle width. The ROC curve was used to establish cut-off values for predicting the prognosis of lateral ventricular in IMVM and IMODVM. The results showed a strong predictive value, allowing us to differentiate between different prognoses of lateral ventricular.

CP abnormalities may affect brain homeostasis, and their impact on central nervous system disorders has recently attracted specialists’ attention [5–8]. Cui [33] revealed that the CP plays a role in intracerebral inflammation during fetal development, influencing cerebral cortex growth. Psychosis spectrum disorders such as schizophrenia and bipolar disorder [7, 9] are related to increased CPV, and an association has been observed between a larger CPV and higher levels of interleukin-6. A study on pediatric autism [8] also noted CPV enlargement. The investigation of the association between CP damage and the observed reduction in CPV on ultrasound imaging constitutes the primary focus of our forthcoming study. Some fetuses with isolated VM have been found to have neurodevelopmental disorders, which affect survival after delivery [20, 22, 34]. Further investigation is necessary to explore the correlation between these altered CPV and neurodevelopmental disorders in fetuses with isolated VM.

### Limitations

This was a pilot observational study conducted at a single hospital center; therefore, bias was inevitable. First, cases of ISVM that could be followed up at birth were rare, and the included fetuses were not present at every gestational week, resulting in an imperfect analysis. Second, the established *z*-score model did not encompass all fetal biometric parameters but only significant parameters associated with the fetal brain. Thirdly, the primary focus of this study lies in the volumetric development of the CP. In our forthcoming research, we aim to broaden the scope of our investigation on fetal CP, encompassing not only a 3D volume analysis but also an assessment of each diameter on 2D ultrasound images. Despite this shortcoming, the study still contributes to our understanding of CP changes in fetuses with isolated VM.

## Conclusions

The changes in CPV among fetuses with isolated VM were assessed using fetal neuro-ultrasound, and these alterations were found to be correlated with the progression of lateral ventricle enlargement. This discovery holds potential implications for prenatal counseling and clinical management strategies in cases involving fetuses with isolated VM. The forthcoming investigation will encompass an exploration of the clinical implications associated with altered CPV in fetuses, specifically focusing on its impact on the neurodevelopment of newborns.

## Supplementary information

ELECTRONIC SUPPLEMENTARY MATERIAL

## Abbreviations

BPD: Biparietal diameter
CP: Choroid plexus
CPV: Choroid plexus volume
CSF: Cerebrospinal fluid
GA: Gestational age
ICC: Intra-class correlation coefficients
IMODVM: Isolated moderate ventriculomegaly
IMVM: Isolated mild ventriculomegaly
ISVM: Isolated severe ventriculomegaly
ROC curve: Receiver operating characteristic curve
SD: Standard deviation
VM: Ventriculomegaly
