# Supplementary material for: Is choroid plexus growth altered in isolated ventriculomegaly on fetal neuro-ultrasound?
Source: Eur Radiol. 2024 Jul 17;35(1):463–73. doi: 10.1007/s00330-024-10966-3 (PMC11632053; doi:10.1007/s00330-024-10966-3)

Is choroid plexus growth altered in isolated ventriculomegaly on fetal neuro-ultrasound?
ELECTRONIC SUPPLEMENTARY MATERIAL

Table 1 STROBE Statement—Checklist of items that should be included in reports of ***cross-sectional studies***

	**Item No**	**Recommendation**	**Page No.**
**Title and abstract**	1	(*a*) Indicate the study's design with a commonly used term in the title or the abstract	1-2
		(*b*) Provide in the abstract an informative and balanced summary of what was done and what was found	1-2
**Introduction**			
Background/rationale	2	Explain the scientific background and rationale for the investigation being reported	4-5
Objectives	3	State specific objectives, including any prespecified hypotheses	5
**Methods**			
Study design	4	Present key elements of study design early in the paper	5
Setting	5	Describe the setting, locations, and relevant dates, including periods of recruitment, exposure, follow-up, and data collection	5-8
Participants	6	(*a*) Give the eligibility criteria, and the sources and methods of selection of participants	6
Variables	7	Clearly define all outcomes, exposures, predictors, potential confounders, and effect modifiers. Give diagnostic criteria, if applicable	7-9
Data sources/ measurement	8*	For each variable of interest, give sources of data and details of methods of assessment (measurement). Describe comparability of assessment methods if there is more than one group	*6-7*
Study size	10	Explain how the study size was arrived at	6
Quantitative variables	11	Explain how quantitative variables were handled in the analyses. If applicable, describe which groupings were chosen and why	6-8
Statistical methods	12	(*a*) Describe all statistical methods, including those used to control for confounding	9
		(*b*) Describe any methods used to examine subgroups and interactions	9
		(*c*) Explain how missing data were addressed	
		(*d*) If applicable, describe analytical methods taking account of sampling strategy	
		(*e*) Describe any sensitivity analyses	
**Results**			
Participants	13*	(a) Report numbers of individuals at each stage of study—eg numbers potentially eligible, examined for eligibility, confirmed eligible, included in the study, completing follow-up, and analysed	10
		(b) Give reasons for non-participation at each stage	10
		(c) Consider use of a flow diagram	Fig 1 and Fig 2
Descriptive data	14*	(a) Give characteristics of study participants (eg demographic, clinical, social) and information on exposures and potential confounders	Table1

		(b) Indicate number of participants with missing data for each variable of interest	10
Outcome data	15*	Report numbers of outcome events or summary measures	10-13
Main results	16	(*a*) Give unadjusted estimates and, if applicable, confounder-adjusted estimates and their precision (eg, 95% confidence interval). Make clear which confounders were adjusted for and why they were included	10-12
		(*b*) Report category boundaries when continuous variables were categorized	
		(*c*) If relevant, consider translating estimates of relative risk into absolute risk for a meaningful time period	
Other analyses	17	Report other analyses done—eg analyses of subgroups and interactions, and sensitivity analyses	12-13
**Discussion**			
Key results	18	Summarise key results with reference to study objectives	13-17
Limitations	19	Discuss limitations of the study, taking into account sources of potential bias or imprecision. Discuss both direction and magnitude of any potential bias	17
Interpretation	20	Give a cautious overall interpretation of results considering objectives, limitations, multiplicity of analyses, results from similar studies, and other relevant evidence	13-16
Generalisability	21	Discuss the generalisability (external validity) of the study results	13-16
**Other information**			
Funding	22	Give the source of funding and the role of the funders for the present study and, if applicable, for the original study on which the present article is based	Disclosure-Paragraph

*Give information separately for exposed and unexposed groups.

Note: An Explanation and Elaboration article discusses each checklist item and gives methodological background and published examples of transparent reporting. The STROBE checklist is best used in conjunction with this article (freely available on the Web sites of PLoS Medicine at http://www.plosmedicine.org/, Annals of Internal Medicine at http://www.annals.org/, and Epidemiology at http://www.epidem.com/). Information on the STROBE Initiative is available at www.strobe-statement.org.

Table 2 Longitudinal reference range for 390 choroid plexus volume from normal fetuses

GA	CP (number)	CPV (mm^3)				
		P5	P10	P50	P75	P90
22	25	407	443.2	531	588	625
23	45	458.2	475.2	548	619.5	656.4
24	23	490	505.6	632	654	678.6
25	27	515	551.2	654	701	716
26	24	553	577	667	748.75	767.5
27	19	613	667	777	813	821
28	23	681.2	726.8	832	852	881
29	31	723.6	749.4	837	881	899.8
30	33	741.8	793.8	861	883	908.8
31	26	778.5	820.1	869.5	912.75	961
32	33	803.4	820.6	890	964.5	1000.8
33	27	812.4	834.8	899	969	1002.8
34	22	815.9	833.3	926.5	975.75	1010
35	32	839.85	863.3	940.5	987	1011.4

GA, gestational age; CP, choroid plexus; CPV, choroid plexus volume;

Table 3. Regression models for prediction of the means and standard deviations (SD)of fetal CPV based on GA or BPD

CPV Parameters	B	Model Derived from Regression Analysis	r^2	p-value	F test
**Means of CPV, mm^3**					
GA, weeks	141.195	$-1690.09 + 141.195 * GA -1.88 * GA^2$	0.99	<.001	521.13
BPD, cm	316.66	$-783.82 + 316.66 * BPD -13.48 *BPD^2$	0.955	<.001	21.17
**SD of CPV, mm^3**					
GA, weeks	0.15	$220.45-9.85 * GA + 0.15 *GA^2$	0.021	0.017	4.10
BPD, cm	2.14	$223.88-37.21 *BPD + 2.14*BPD^2$	0.046	<.001	9.26

CPV, choroid plexus volume; GA, gestational age; BPD, biparietal diameter

Table 4 Spearman correlation analysis was performed to investigate the relationship between independent variables (lateral ventricular width, birth weight) and dependent variables (CPV, CPV z-scores) in both the control group and study group

**Dependent variables**	Correlation Coefficient	P value	**Dependent variables**	Correlation Coefficient	P value
**Spearman correlation analysis with lateral ventricular width**					
control group			study group		
CPV	0.432	< .001	CPV	-0.342**	< .001
CPV z-scores against GA	0.267	< .001	CPV z-scores against GA	-0.476**	< .001
CPV z-scores against BPD	0.285	< .001	CPV z-scores against BPD	-0.523**	< .001
**Spearman correlation analysis with with birth weight**					
control group			study group		
CPV	-0.071	0.163	CPV	-0.124	0.148
CPV z-scores against GA	0.07	0.168	CPV z-scores against GA	-0.124	0.156
CPV z-scores against BPD	0.033	0.512	CPV z-scores against BPD	-0.127	0.139

CPV, choroid plexus volume; GA, gestational age; BPD, biparietal diameter; p < 0.01 means there was a correlation between parameters.

Table 5 Line analysis was performed in control group and study group

**Dependent variables**	**Independent variables**	**Correlation Coefficient**	**t**	**95% CI**	**R^2**
The control group					
CPV	lateral ventricular width	53.705**	-9.261	42.339 ~ 65.070	0.181
linear regression model：CPV（mm^3）=386.275 + 53.705*lateral ventricular width					
CPV z-scores against GA	lateral ventricular width	0.204**	5.449	0.131 ~ 0.277	0.071
linear regression model：CPV Z-scores (against GA)=-1.512 + 0.204*lateral ventricular width					
CPV z-scores against BPD	lateral ventricular width	0.214**	5.628	0.140 ~ 0.289	0.075
linear regression model：CPV Z-scores (against BPD)=-1.532+ 0.214*lateral ventricular width					
The study group					
CPV	lateral ventricular width	-69.750**	-4.764**	-98.448 ~ -41.052	0.144
linear regression model：CPV（mm^3）= 1595.250-69.750*lateral ventricular width					
CPV z-scores against GA	lateral ventricular width	-1.360**	-7.764	-1.704 ~ -1.017	0.309
linear regression model：CPV Z-scores (against GA)= 15.913-1.360*lateral ventricular width					
CPV z-scores against BPD	lateral ventricular width	-1.479**	-8.78	-1.809 ~ -1.148	0.363
linear regression model：CPV Z-scores (against BPD)=17.017-1.479*lateral ventricular width					
IMVM fetuses with regressive lateral ventricular					
CPV z-scores against GA	Time interval	0.313**	5.799	0.207 ~ 0.418	0.505
linear regression model：CPV Z-scores (against GA)= -7.710 + 0.313*Time interval					
CPV z-scores against BPD	Time interval	0.302**	6.351	0.209 ~ 0.395	0.55
linear regression model：CPV Z-scores (against BPD)=-7.510 + 0.302Time interval					

*CPV, choroid plexus volume; GA, gestational age; BPD, biparietal diameter;**means p < 0.01.*

Fig 1 A QQ plot of CPV z-scores against GA and BPD

CPV, choroid plexus volume; GA, gestational age; BPD, biparietal diameter

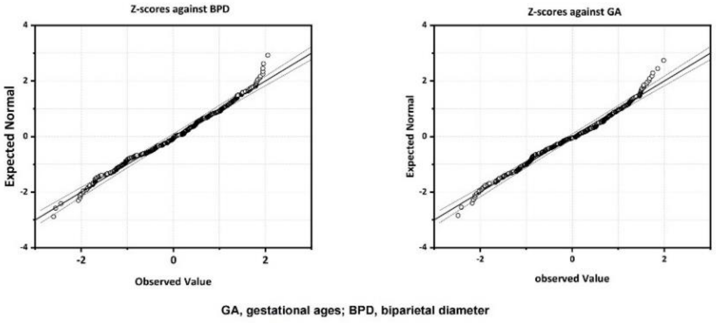

Supplement: Supplementary file 1 — ELECTRONIC SUPPLEMENTARY MATERIAL [file 330_2024_10966_MOESM1_ESM.pdf]
